# How can ethical leadership increase employees' bootlegging innovation behavior in China?: a serial mediation model of psychological wellbeing and psychological entitlement

**DOI:** 10.3389/fpsyg.2025.1506906

**Published:** 2025-03-04

**Authors:** Junzhu Zhang, MyeongCheol Choi, Kaiyuan Wang, Hann Earl Kim

**Affiliations:** Department of Business, Gachon University, Seongnam, Republic of Korea

**Keywords:** ethical leadership, psychological wellbeing, psychological entitlement, bootlegging innovation behavior, serial mediation model

## Abstract

Bootlegging innovation behavior poses challenges to organizational management but significantly contributes to development and innovation. The relationship between leadership style and bootlegging innovation behavior is particularly noteworthy. Ethical leadership often instills a sense of reliability and integrity in employees, fostering trust and reciprocity, which benefits both leaders and the organization. To investigate how ethical leadership enhances employees' willingness to innovate and engage in innovative behavior, this study collected survey data from 382 private-sector employees in southeast China. The findings revealed a positive correlation between ethical leadership and bootlegging innovation behavior. Furthermore, psychological wellbeing and psychological entitlement were found to mediate the relationship between ethical leadership and bootlegging innovation behavior. The integrity and inclusiveness demonstrated by ethical leadership help employees feel valued, enhancing their psychological wellbeing. This, in turn, increases their psychological entitlement and willingness to realize their self-worth, making them more inclined to put innovative ideas into practice—even without explicit authorization. The key contribution of this study lies in identifying the chain-mediating effect of psychological wellbeing and psychological entitlement in the relationship between ethical leadership and bootlegging innovation behavior. By exploring this complex and nuanced process, the study provides actionable recommendations for fostering bootlegging innovation behavior among employees. It also broadens the research scope of bootlegging innovation and offers fresh perspectives for future research endeavors.

## Introduction

Innovation is an important way to help companies maintain and expand their competitive advantage in the process of organizational development and change (Tushman and O'Reilly, 2002). The growth and profitability of a business are closely linked to the spirit of innovation in the organization (Antoncic, 2007). Organizational innovation is triggered by both a deliberate top-down management approach and a bottom-up burst process (Grant, 2003; Globocnik et al., 2022). The spirit of innovation depends on the individual behavior of employees in the organization, and they can innovate with or without formal authorization from management (Krueger and Buchwald, 2023). Therefore, it is important to understand how to promote innovative behavior among members of organization (Krueger et al., 2000). One form of innovation that deserves attention is that employees sometimes ignore the organization's institutional rules and compliance procedures to articulate their original ideas (Kanter, 2006). This is called bootlegging innovation behavior, which is a specific form of innovation in which researchers completely ignore management directives, act covertly, and decide on their own to invest in company resources and pursue inventive ideas (Augsdorfer, 2005). Through this bottom-up, unplanned creative activity, innovative ideas that benefit the organization are generated and elaborated upon without authorization (Augsdorfer, 2005; Globocnik et al., 2022). From an individual perspective, bootlegging provides more freedom to explore uncharted territory, giving employees a greater competitive advantage over colleagues who do not engage in this behavior (March, 1991). From an organizational perspective, this behavior enables individuals to develop and innovate more and create value for the organization (Criscuolo et al., 2014). Bootlegging can help organizations overcome innovation inertia, identify and exploit opportunities that are not considered in the organization's pre-planned strategies, and promote innovation at a lower additional cost (Koch and Leitner, 2008). Therefore, it is necessary to discuss the causes of employee bootlegging innovation behavior, pay attention to its importance in the organization, and promote it to improve the competitive advantage of enterprises and promote their sustainable development.

According to previous research, the factors influencing employee bootlegging innovation behavior include leadership behavior, individual job design, and personal factors. In organizations, negative leadership styles, such as abusive supervision, can lead to decreased job satisfaction (Wang et al., 2020), less work engagement (Ampofo, 2021), and reduced bootlegging innovation behaviors (Wang X. L. et al., 2023). Incentives for entrepreneurial self-efficacy, strategic autonomy, and innovative achievements have led to increased employee bootlegging (Globocnik and Salomo, 2015). In terms of individual employee factors, the risk propensity of individual employees directly affects bootlegging innovation behavior, especially when managers encourage innovation (Globocnik, 2019). Studies have shown that employees need to feel empowered and motivated to take entrepreneurial action (Morrison and Phelps, 1999; Goldsby et al., 2006). Thus, leadership styles related to employee motivation to engage in innovation (Schuckert et al., 2018), psychological wellbeing (He et al., 2019), and psychological entitlement (Mao et al., 2023) can explain the emergence of bootlegged innovation behavior. This study suggests that a positive leadership style is a key factor in increasing employees' specific innovation behaviors, and ethical leadership is directly related to bootlegging innovation behaviors. Ethical leadership refers to demonstrating normative and appropriate behavior through personal and interpersonal actions and promoting this to followers through two-way communication, reinforcement, and decision-making (Brown et al., 2005). Ethical leadership has two components: moral and ethical (Khan and Javed, 2018). Ethical leadership places high value on personal morality, deliberately shapes the role of ethical behavior, and creates accountability by linking employees' ethical behavior to the organizational performance management system and thus to rewards and punishments (Brown and Treviño, 2006; Khan and Javed, 2018).

Regarding the influence mechanism of leader behavior on employees' bootlegging innovation behavior, scholars have studied the indirect effects on behavior of factors such as paradoxical leadership (Yang et al., 2024), abusive supervision (Wang X. L. et al., 2023), and ethical leadership from the perspective of gender similarity (Li et al., 2021), but they have not been explored from other perspectives. When investigating complex social phenomena or behaviors, a single perspective may be insufficient to fully capture their multidimensional characteristics (Canter, 2012). Adopting multiple perspectives allows for a more comprehensive understanding of the relationship between ethical leadership and bootlegging innovation behavior, contributes to the development of a more robust knowledge framework, and enhances the external validity and practical applicability of research findings. For instance, a sociological perspective on ethical leadership may focus on the dynamics between leaders and organizational members (Neves and Story, 2015), whereas an economic perspective might emphasize the effects of piracy innovation on market competition (Bradley and Kolev, 2023). In this context, the present study adopts an employee psychological perspective. Specifically, it seeks to address the following three research questions: Is bootlegging innovation behavior a form of disregard for managerial authority (Augsdorfer, 2005), and does ethical leadership directly lead to this behavior? How does ethical leadership affect employees' bootlegging innovation behavior? How does ethical leadership affect bootlegging innovation behavior from the perspective of employee psychology?

Previous research has suggested that the psychological wellbeing that arises or increases when employees are influenced by ethical leadership leads to persistent positive perceptions of themselves (De Roeck and Farooq, 2018; Ilyas et al., 2023). In such cases, employees' sense of entitlement and worthiness is enhanced (Ciulla, 2004; Lennick and Kiel, 2011). Psychological entitlement increases employees' willingness to actively participate in a company's activities, which is a phenomenon that often occurs when employees are aware of positive feedback and fairness within the organization (Galvin et al., 2015; Kurth, 2023). When creative proposals are rejected, employees remain positive about themselves and the organization and do not abandon their ideas but instead go underground (Criscuolo et al., 2014; Mainemelis and Sakellariou, 2023). Therefore, this study suggests that psychological wellbeing and psychological entitlement mediate the relationship between ethical leadership and bootlegging innovation behavior.

This study considers ethical leadership as an antecedent of bootlegging innovation behavior and argues that the former has a positive impact on the latter. This is one of the first studies to explore how positive leadership promotes bootlegging innovation behaviors among employees. There have been many studies that have shown that ethical leadership has a positive impact on employees' innovative behavior and performance (Ahmed Iqbal et al., 2020; Ullah et al., 2021; Hoang et al., 2023). By focusing on positive ethical leadership behavior, this study examines the influence mechanism of ethical leadership on employees' bootlegging innovation behavior. This study argues that psychological wellbeing and psychological entitlement mediate the relationship between ethical leadership and employees' bootlegging innovation behavior, and explores the sequential mediation role of psychological wellbeing and psychological entitlement. This study responds to the call of Globocnik et al. (2022) to consider bootlegging innovation behavior as a trend from an organizational perspective and contributes to further expanding the field of research on bootlegging innovation behavior.

## Theory and hypotheses

### Ethical leadership and bootlegging innovation behavior

This study argues that ethical leadership, as a fair and tolerant leadership style, directly impacts employees' bootlegging innovation behavior. Ethical leadership is defined as demonstrating normatively appropriate behavior through personal actions and interpersonal relationships, and promoting this conduct to followers through two-way communication, reinforcement, and decision-making (Brown et al., 2005; Shiundu, 2024). This emphasizes that ethical leadership is a perceptible behavior designed to benefit employees (Den Hartog, 2015). Brown et al. (2005) argued that the core components of ethical leadership include acting fairly, allowing opinions to be heard, and rewarding moral behavior. Scholars have also considered sustainability and social or broader rights issues (Kalshoven et al., 2011). From the perspective of social influence, some scholars believe that ethical leadership is the process of using group activities to achieve goals in a socially responsible way (Den Hartog and De Hoogh, 2009; Aronson, 2001). In this approach, ethical leaders are driven by moral beliefs and caring values, and their goal is to make their actions and judgments beneficial to followers, organizations, and society (Kalshoven et al., 2011; Den Hartog, 2015; Shiundu, 2024). Based on the social exchange theory (Cropanzano and Mitchell, 2005), the reciprocal obligation model arises from a relationship that develops through a series of mutual exchanges (Masterson et al., 2000). Over time, the norm of reciprocity causes followers to repay fair and caring treatment from ethical leaders by demonstrating the desired behavior (Walumbwa et al., 2011; Piecek, 2023), which also involves a wide range of constructive behaviors to the work team or organization (Kalshoven et al., 2013; Den Hartog, 2015). This study suggests that under such circumstances, ethical leadership has a positive impact on employees' bootlegging innovation behavior and promotes its generation.

Bootlegging innovation behavior refers to the actions of employees who engage in pre-development activities without official authorization or supervisory oversight. These individuals often utilize their own resources, which go unnoticed during the exploration phase, and circumvent formal communication channels when promoting their ideas (Howell and Higgins, 1990). Such behavior occurs “underground,” making it challenging to control through traditional management practices (Globocnik and Salomo, 2015; Yang et al., 2024). It embodies a paradoxical nature, where legitimate ends are pursued through potentially unlawful means (Criscuolo et al., 2014). Moreover, bootlegging innovation behavior represents a broader concept that encompasses, but is not limited to, creative deviance (Yang et al., 2024). Creative deviance specifically describes situations in which employees persist in developing new ideas despite explicit directives from leaders to cease such activities. Bootlegging innovation behavior broadly captures the phenomenon of employees initiating and advancing new ideas without organizational approval or in direct violation of managerial orders (Lin et al., 2016; Globocnik, 2023). Examining the antecedents of bootlegging innovation behavior offers insights into a wider spectrum of factors that influence employees' independent generation and development of innovative ideas (Yang et al., 2024). This study posits that ethical leadership positively influences employees' propensity for bootlegging innovation behavior. Employees are inclined to engage in constructive activities, such as bootlegging innovation, as a form of reciprocation for the fair and supportive treatment provided by ethical leaders (Kalshoven et al., 2013; Den Hartog, 2015). Furthermore, this behavior is more likely to emerge when leaders adhere to formal rules while tolerating certain employee violations (Criscuolo et al., 2014; Yang et al., 2024). The fair and supportive conduct demonstrated by ethical leadership fosters a sense of psychological safety and wellbeing among employees, enabling them to challenge conventional norms without excessive pressure to conform to established rules. In this context, ethical leadership cultivates a positive and supportive environment for bootlegging innovation by encouraging value-oriented behavior and enhancing team trust and empowerment. Rather than suppressing bootlegging innovation through rigid enforcement of rules, ethical leaders create conditions where such behaviors can thrive. While maintaining organizational norms is crucial for ensuring stability and preventing chaos, the tacit tolerance of underground innovation pursuits may yield substantial benefits for leaders and organizations if these initiatives evolve into successful products (Criscuolo et al., 2014). Employees often perceive that leaders will tolerate their bootlegging activities as they ultimately benefit the team or organization (Masoudnia and Szwejczewski, 2012; Yang et al., 2024). The intrinsic motivation to demonstrate self-worth through innovative behavior drives employees' enthusiasm and proactivity, making bootlegging innovation an appealing strategy, especially in organizations with limited resources or less well-defined rules. Additionally, the risk-tolerant nature of ethical leadership further encourages employees to adopt bootlegging innovation behaviors, despite their controversial nature. Moreover, ethical leadership does not involve the rigid enforcement of organizational rules but instead emphasizes ethics and values (Lemoine et al., 2019). This leadership style encourages employees to critically reflect on the rationality of existing rules and procedures, fostering and enhancing their critical thinking. Such reflection may prompt employees to adopt unconventional, rule-defying approaches to innovation. This study argues that ethical leadership can strengthen employees' intrinsic motivation, encouraging them to act based on specific circumstances and focus on solving problems creatively. This approach is more likely to lead employees to break rules constructively while adhering to moral principles, thereby supporting bootlegging innovation behaviors, rather than merely conforming to formal compliance. Therefore, Hypothesis 1 is proposed:

*H1: Ethical leadership has a positive effect on bootlegging innovation behavior*.

### Mediating effect of psychological wellbeing

Psychological wellbeing is a combination of feeling good and functioning effectively, meaning that life is going well. When negative emotions are extreme or persist for a long time and interfere with one's daily life, psychological wellbeing is compromised (Huppert, 2009). Psychological wellbeing is defined as a state in which an individual was able to realize their abilities, cope with normal life stresses, work productively, and contribute to the community (World Health Organization, 2001). People with high levels of subjective wellbeing tend to have a more attributive approach to self-improvement and potential than those with low subjective wellbeing, suggesting that positive emotions can lead to favorable cognition and produce pleasing emotions (Ryan and Deci, 2001). Studies using emotion-inducing techniques have shown that participants with positive mood states have a wider focus (Gasper and Clore, 2000), generate more ideas (Fredrickson and Branigan, 2005), and think more creatively and flexibly (Bless et al., 1992). Positive emotions lead to good behavior and enhanced cognitive abilities, which, in turn, stimulate upbeat emotions (Fredrickson and Joiner, 2002). Huppert (2009) studied the factors influencing psychological wellbeing, which are personality, demographic characteristics, socioeconomic status, and other drivers. In terms of personality, extraversion is associated with positive emotional styles (Costa and McCrae, 1980). The effect of neuroticism on psychological wellbeing is fully mediated by psychological distress and disappears completely when psychological distress is controlled (Huppert, 2009). In terms of demographic characteristics, the effect of personality on psychological wellbeing is less pronounced (Helliwell, 2003), and age has a U-shaped relationship with psychological wellbeing, as rated using a single wellbeing measure (e.g., life satisfaction; Blanchflower and Oswald, 2008). Higher income and socioeconomic status are associated with elevated happiness (Ryff and Singer, 1998), but this effect wanes with increased income levels (Huppert, 2009). The degree of national income inequality is negatively correlated with psychological wellbeing (King et al., 2024; García-Sánchez et al., 2024), and a few studies have found a negative correlation between educational attainment and psychological wellbeing (Fagg et al., 2008). An increase in the level of depression resulting from higher educational attainment may indicate work-related stress in occupations that require a degree (Chevalier and Feinstein, 2006). Unemployment is also associated with psychological wellbeing (Evans and Repper, 2000), as people who are relatively happy at first become unhappy after unemployment (Lucas et al., 2004). Other factors influencing psychological wellbeing include conscious controllable activities such as behavior, cognition, and motivation (Lyubomirsky et al., 2005; Sheldon and Lyubomirsky, 2006).

In the field of organizational behavior, there has been a lot of literature on positive psychology and positive organizational behavior that suggests that positive work experiences affect the psychological wellbeing of individuals (Ryff and Keyes, 1995; Ryff and Singer, 2002), which in turn influences attitudes toward the organization (Luthans, 2002). According to social exchange theory (Cook and Emerson, 1987), when employees feel they are treated fairly by ethical leaders, they have a stronger rapport with their leaders, see leaders as role models, and have respect and gratitude for their leaders. Consequently, employees expect a positive impact on bootlegging innovation behavior by making a favorable return for their efforts that are beneficial to the leader or organization. This study posits that ethical leadership demonstrates care for employees' individual needs, growth, and wellbeing through fair decision-making and respectful treatment. Such fairness, trust, and a supportive environment reduce employees' stress and anxiety. The sense of respect and trust experienced by employees enhances their feelings of belonging and self-worth, leading to greater happiness and fulfillment in their work. When leaders have a caring and inclusive attitude toward employees, employees have a positive psychological state and a higher degree of mental health; therefore, they are more willing to devote their time and energy to bootlegging innovation behavior for the organization develop (Sison, 2003; Li et al., 2022).

In addition, ethical leadership serves as a role model by upholding high ethical standards and guiding employees to pursue greater goals, thereby directly enhancing their wellbeing (Yang, 2014). Employees experience significantly greater happiness when they perceive their work as meaningful and fulfilling (Charles-Leija et al., 2023). When employees come up with creative ideas that are beneficial to the organization to solve work problems, they perceive that the leader will tolerate their behavior that deviates from the organization's norms (Masoudnia and Szwejczewski, 2012; Yang et al., 2024). Ethical leadership fosters a work environment that encourages experimentation by minimizing harshness. This tolerance for failure and mistakes, coupled with the recognition of effort, helps reduce anxiety and enhance employees' psychological wellbeing. This study argues, there is less pressure to engage in bootlegging innovation behaviors, and psychological wellbeing is better, which may increase employees' willingness to go underground to implement innovative ideas.

Psychological wellbeing, as a positive psychological state that fosters cognitive flexibility and creative thinking, provides emotional support for employees to explore unconventional ways of thinking and pursue innovative breakthroughs (Helzer and Kim, 2019; Muñoz-Pascual and Galende, 2020). Employees with high psychological wellbeing show more positive attitudes and respond better to various situations in their lives than do those with low psychological wellbeing (Ryff and Keyes, 1995). Psychological wellbeing enhances employees' focus on problem-solving, fosters a sense of accomplishment and self-worth at work, and stimulates their intrinsic motivation. In this situation, employees are still more productive when innovative ideas are rejected because of optimism and positivity about the outcome of their behavior (Aggarwal-Gupta et al., 2010), resulting in bootlegging innovation behavior. Psychological wellbeing reduces employees' sensitivity to rules and constraints (Erkutlu and Chafra, 2016). Employees with higher levels of happiness tend to have greater self-confidence and a stronger sense of security, which makes them more likely to focus on the potential benefits of innovation rather than excessively worrying about the risks associated with rules (Guberina et al., 2023). Since bootlegging innovations often involve circumventing traditional rules, wellbeing increases the likelihood of engaging in such innovations. Furthermore, employees with high psychological wellbeing are more inclined to share ideas, exchange resources, and collaborate with others to innovate (Berraies et al., 2020). Given that the success of bootlegging innovation is closely tied to collaboration among team members, we argue that psychological wellbeing plays a key role in driving the implementation and success of this collaborative process. Therefore, this study explores the mechanism through which ethical leadership influences employees' bootlegging innovation behavior through psychological wellbeing. We argue that ethical leadership enhances employees' psychological well-being through caring, supportive, fair, and goal-oriented leadership behaviors. This positive psychological state, in turn, provides the necessary psychological safety and emotional foundation for engaging in pirated innovation behaviors. It stimulates employees' creativity and willingness to challenge traditional norms, fostering the emergence and development of pirated innovation within organizations. Based on this logic, Hypothesis 2 is proposed:

*H2: Psychological wellbeing positively mediates the relationship between ethical leadership and bootlegging innovation behavior*.

### Mediating effect of psychological entitlement

Psychological entitlement refers to the phenomenon in which individuals consistently believe that they deserve generous rewards and treatment with little regard to their actual qualities or performance levels (Harvey and Martinko, 2009), which has been of great concern to managers (Harvey and Harris, 2010). Psychological entitlement is not necessarily based on a genuinely fair exchange (Harvey and Martinko, 2009; Kim and Chung, 2023). Previous scholars have explored the definition of entitlement from different perspectives. Naumann et al. (2002) collated and reviewed these studies, arguing that all fields view rights as related to what individuals believe they deserve. In the legal world, rights are seen as something conferred by law and cannot be removed without due process (Black et al., 1999; Pennington, 2023). An individual's entitlement is seen as a dichotomy in which there are only two possibilities for property or right to belong to someone or not (Naumann et al., 2002; Krivenko, 2023). In the field of philosophy, Nozick and Nagel (1974) proposed the entitlement theory, which argues that people's past circumstances can create different rights. Individuals have a fundamental right to liberty, life, and health against harm and control over personal property (Naumann et al., 2002; Roberts-Cady, 2024). In economics, entitlement is conceptualized as an economic good that can be exchanged for other goods and services (Thomas and Wu, 2006; Durán-Sandoval and Uleri, 2023). In the field of management, employees' perceptions of entitlement influence whether they perceive change as psychological contract violation, which feels more direct and intense than the perception of unmet expectations or unfairness (Robinson and Rousseau, 1994; Rodwell and Ellershaw, 2024). Naumann et al. (2002) defined an employee's perceived entitlement as the compensation that an individual is expected to receive after participating in an employment relationship, arguing that perceived entitlement is based on an unbalanced assessment of reciprocity. Individuals with a strong sense of entitlement expect rewards and compensation from the organization without the need to reciprocate by achieving high levels of performance (Harvey and Martinko, 2009). Entitlement-conscious individuals often expect important events to go their way (Snow et al., 2001; Schwarz et al., 2023). Similarly, high levels of psychological entitlement can lead to corruption and overly self-centered behavior among organizational leaders (Levine, 2005; De Clercq, 2023).

In a team managed by ethical leaders, employees feel that the organizational culture is inclusive, genuine, fair, and ethical. In this environment, employees often have a positive view of the organization and themselves. Individuals with power consistently have a positive view of themselves as an important aspect of psychological entitlement (Snow et al., 2001; Lin et al., 2023). Employees with high subjective rights tend to have a high opinion of themselves and carry out their work with extremely inflated needs and expectations (Crampton and Hodge, 2009). In addition, such people believe that they are consistently more worthy than others (Campbell et al., 2004). Individuals with high levels of psychological entitlement are selfish in interpersonal relationships and exhibit relatively low levels of empathy, perspective and respect (Campbell et al., 2004; Chen et al., 2024). The stability of psychological entitlement is not affected by the passage of time and may be secure in a variety of situations (Campbell et al., 2004). Snow et al. (2001) defined psychological entitlement as a personality construct that can have a comprehensive impact on an individual's thoughts and behaviors.

Previous studies have shown that leadership style is an important organizational environmental factor for predicting employees' psychological entitlement. For example, Si W. et al. (2023) conducted research based on social comparison theory, and found that servant leadership may lead employees to take it for granted that the leader should prioritize themselves, and that employees may become psychologically powerful and have increased psychological entitlement. When negative feedback from leaders is weak, servant leadership triggers a sense of psychological entitlement among followers with a high degree of Machiavellianism (Gao and Liu, 2023). In addition, a high level of leader humility elevates followers' perceptions of themselves, manifesting as a higher level of pride, which leads to a sense of psychological entitlement (Bahmannia et al., 2023). In addition, there is an association between the behavior of individual employees and psychological entitlement; for example, unethical pro-organizational behavior produces psychological entitlement because it involves doing something positive for the organization (Jiang et al., 2023).

According to previous research, psychological entitlement influences employees' thoughts and behaviors. The entitled may be drawn to conspiracy theories as a defensive mechanism against threat to their self-concept, where a conspiracy is a shadowy secret piece of information accessible only to a few “insiders” (Neville et al., 2024). Conspiracy theories provide followers with a sense of exaggerated importance (Van Prooijen, 2022). In addition, employees with stronger psychological entitlement may be more willing to engage in unethical pro-organizational behavior because they want to achieve a higher status (Wang H. et al., 2023). The reasons behind such behaviors, which may objectively lead to organizational benefits, may not necessarily align with the organization's interests. Individuals with a high sense of psychological entitlement may act selfishly, not driven by a desire to contribute to the organization's development, but rather by personal gain. Based on attribution theory, Harvey and Martinko (2009) argued that the tendency of empowered employees to attribute desirable outcomes to internal factors is expected to exacerbate this effect, leading to the formation of attitudes in employees' favor, while arguing that organizations have little responsibility for positive outcomes. In this context, employees evaluate their innovative ideas and abilities well, and believe that they should implement innovative behaviors regardless of whether the organization supports or empowers them or not. Because this type of innovation is not likely to be explicitly authorized or supported by the organization, employees with a high level of psychological entitlement tend to believe that the organization is not responsible for the success of their innovation. Therefore, this study argues that ethical leadership fosters a harmonious and inclusive work environment while serving as a role model. In such an environment, employees' self-confidence is enhanced, and they develop a high opinion of themselves, often leading them to acknowledge—and potentially overestimate—their value within the organization. However, individuals with high levels of psychological entitlement tend to place significant emphasis on personal autonomy (Crampton and Hodge, 2009; Chen et al., 2024), which may conflict with the role models and rules promoted by ethical leadership. We contend that this divergence is a key factor in the emergence and development of bootlegging. Individuals with a strong sense of psychological empowerment are more likely to implement their innovative ideas, driven by the desire for self-actualization or personal gain, even when these ideas diverge from the principles emphasized by ethical leadership.

Previous studies have indicated that high levels of psychological entitlement may create a perceived sense of wellbeing due to the support provided by the organization. However, this psychological tendency can diminish employees' sense of responsibility toward the organization (Feather, 1999). Employees with high psychological entitlement, when believing their innovation benefits the organization, may lack the motivation to actively contribute to the generation of new outcomes and instead focus on completing only the bare minimum. Conversely, employees with high psychological entitlement often prioritize their individual goals and interests, leading them to exhibit strongly self-directed behaviors (Campbell et al., 2004). This tendency enables them to actively explore creative solutions beyond their assigned tasks for personal benefit. Additionally, ethical leadership fosters a sense of belonging and trust within the organization (Avolio et al., 2009). We propose that this trust can mitigate the potential negative effects of psychological entitlement, creating a supportive and secure team environment. Such an atmosphere can redirect the influence of psychological entitlement toward positive innovative behaviors. As time passes, employees in this team atmosphere are increasingly able to feel the care and fairness of the leader, and are expected to develop according to their wishes, thus producing underground bootlegging innovation behavior. In addition, ethical leadership highlights the value of employee contributions (Treviño et al., 2000). This study suggests that the behaviors and principles of ethical leadership can amplify the self-motivational effects of psychological entitlement. Employees with high levels of psychological entitlement are likely to feel that their innovative efforts will be recognized and respected within this incentivizing framework, thereby fostering the emergence of self-directed bootlegging innovation behaviors. Based on the above, we hypothesize:

*H3: Psychological entitlement positively mediates the relationship between ethical leadership and bootlegging innovation behavior*.

### The chained mediation role of psychological wellbeing and psychological entitlement

According to previous research, psychological wellbeing increases with promoted ethical leadership, which leads employees to have a positive perception of themselves (De Roeck and Farooq, 2018; Ilyas et al., 2023). An increase in an employee's positive self-perception is often accompanied by a rise in their sense of meaningfulness and self-efficacy (Pierce and Gardner, 2004). As a result, employees' psychological entitlement increases as their sense of deserving grows (Ciulla, 2004; Lennick and Kiel, 2011). Individuals in this state are concerned about what others think of them, place great value on gaining recognition and praise from others (Rose and Anastasio, 2014; Schwarz et al., 2023). Such individuals want to maintain a positive self-image and gain a high status in the organization, and are willing to take shortcuts to achieve this (Lee et al., 2019; Valencia Casallas and Barreto-Galeano, 2023). The pathways to attaining status can be divided into prestige-based status and dominance-based pathway (Maner and Case, 2016; Jensen, 2023). The path to prestige-based status involves attaining status through sharing expertise, possessing skills, or achieving socially valuable achievements, promoting respect and voluntary obedience from others. The dominance-based pathway, on the other hand, involves the acquisition of fear through intimidation and coercion (Lange et al., 2019; Maner and Hasty, 2023). Employees who feel encouraged and affirmed by their leaders with psychological wellbeing will perceive themselves as capable of doing their job and will want to prove themselves more and achieve a higher status (Reave, 2005; Si X. et al., 2023). Psychological wellbeing, as a positive state of mind, does not necessarily equate to complete satisfaction with the status quo. Instead, this positive state can enhance employees' confidence and willingness to explore new opportunities and challenges. According to Maslow's (1987) hierarchy of needs theory, when individuals experience happiness, lower-level needs, such as security and belonging, are likely satisfied, leading them to focus on higher-level goals, such as self-actualization and fulfillment. We argue that when employees experience psychological wellbeing, their sensitivity to social status may increase, prompting them to compare themselves to others with higher status, thus generating motivation to pursue greater status. As psychological wellbeing boosts employees' confidence—or even leads to self-inflation—they may come to believe that higher status, along with corresponding resources, rights, and respect, is something they deserve, which can increase their sense of psychological entitlement. In the process of competing for status, employees may begin to expect additional benefits, such as resources and rights, further reinforcing the belief that they deserve more privileges (Ridgeway, 2014; Valencia Casallas and Barreto-Galeano, 2023). Therefore, this study contends that psychological wellbeing and dissatisfaction with the status quo are not contradictory but rather complementary sources of motivation for employees' growth. Wellbeing provides positive psychological resources, while dissatisfaction drives employees to seek breakthroughs and innovations.

In addition, employees' psychological entitlement increases their willingness to actively participate in the company's activities, a phenomenon that often occurs when employees are aware of positive feedback and fairness within the organization (Galvin et al., 2015; Kurth, 2023). Therefore, psychological entitlement can positively impact employees' bootlegging innovation behavior (Lyu et al., 2022; Bennett et al., 2024). These results suggest that ethical leadership can increase employees' psychological wellbeing and psychological entitlement levels, and ultimately have a series of positive effects on bootlegging innovation behavior.

Therefore, according to the social learning theory, psychological wellbeing and psychological entitlement may positively mediate the relationship between ethical leadership and bootlegging innovation behavior. In other words, this study hypothesizes that ethical leadership increases employees' psychological wellbeing, psychological entitlement, and bootlegging innovation behavior.

When there is no link between behavior and punishment that is not authorized by the organization or leader, it may be caused by the inclusion and care of the ethical leader (Bedi et al., 2016; Amore et al., 2023), leaders care for employees to relieve their anxiety when they engage in bootlegging innovation behaviors (Lin et al., 2016; Qu et al., 2023). Employees observed that innovative behaviors were rewarded and they received positive feedback (De Jong and Den Hartog, 2007). In this case, employees appreciate their leaders' guidance and expect something in return that boosts their self-confidence (Hougaard and Carter, 2018; Covington, 2023). This belief in one's ability to bring innovation and change for the organization's development increase employees' motivation to work and innovate (Howell, 2005; Wang and Xie, 2023), thereby positively influencing bootlegging innovation behavior.

Employees learn by observing the behavior of leaders and other members, and they model this behavior, especially those who find it valuable or beneficial (Abiodun, 2010; Liu et al., 2023). A harmonious working environment is created in the team managed by the ethical leader (Bello, 2012; Yuan et al., 2023). Employees find that bootlegging innovation behaviors tend to benefit the organization, and employees who have previously engaged in such behaviors receive positive feedback and rewards (Augsdorfer, 2005; Huang et al., 2022). As a result, they promote imitation, want to be recognized, and think they deserve recognition, ultimately having a positive impact on bootlegging innovation behavior.

Ethical leadership's nurturing and fair treatment enhances employees' self-efficacy, and employees with high self-efficacy are more likely to try and persist in completing a certain task (Ren and Chadee, 2017; Uppathampracha and Liu, 2022). Employees believe that they can successfully complete bootlegging innovation behavior (Li et al., 2021; Li and Ye, 2021; Globocnik, 2023), which is likely to be beneficial to the organization, leaders, and themselves (Augsdorfer, 2005; Yang et al., 2024; Shang, 2024). This behavior objectively rewards the leader's care, affection, and ability to receive praise from others, while subjectively fulfilling the employee's expectation of maintaining a positive self-image and gaining high status within the organization. Therefore, Hypothesis 4 is proposed:

*H4: Psychological wellbeing and psychological entitlement mediate the relationship between ethical leadership and bootlegging innovation behavior in a chained mediation model*.

**Figure 1:**
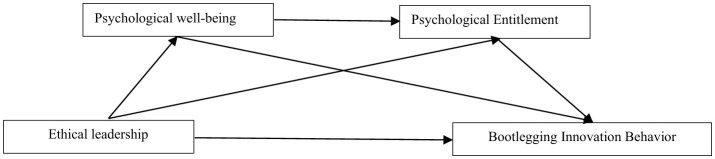
Research model.

The research model of this study is shown in Figure 1.

## Methods

### Sample and data collection

The survey subjects of this study were eight regional and national property companies from Henan, Jiangsu, and Zhejiang provinces in China. A total of 500 questionnaires were distributed and 392 were returned, with a response rate of 78%. After excluding invalid questionnaires, 382 valid questionnaires were used.

### Measures

SPSS, Stata, and M-plus were used for data analysis. M-plus was used for the confirmatory factor and path analyses.

The questionnaire used a 7-point Likert scale, where 1 = strongly disagree, 2 = somewhat disagree, 3 = do not strongly agree, 4 = average, 5 = generally agree, 6 = strongly agree, and 7 = completely agree.

Ethical leadership is measured using five items from Brown et al. (2005). This scale has good reliability and validity and has been adopted by a large number of empirical studies in China and abroad. Sample items include, “Listens to what employees have to say,” “Makes fair and balanced decisions,” and “Sets an example of how to do things the right way in terms of ethics” (Cronbach's alpha = 0.928).

Psychological wellbeing was measured using 10 items from Ryff and Keyes (1995). Sample items include, “I have good interpersonal relationships” and “I think I have continuous growth,” etc. (Cronbach's alpha = 0.886).

Psychological entitlement was measured using five items taken from Campbell et al. (2004). Sample items include, “I honestly feel I'm just more deserving than others” and “I feel entitled to more of everything,” etc. (Cronbach's alpha = 0.850).

Bootlegging innovation behavior was measured using four items from Criscuolo et al. (2014). Sample items include, “I have the flexibility to work my way around my official work plan, digging into new potentially valuable business opportunities,” “My work plan does not allow me the time to work on anything other than the projects I have been assigned to,” “I am running several pet projects that allow me to learn about new areas,” and “I proactively take time to work on unofficial projects to seed future official projects.” Among these, the statement “My work plan does not allow me the time to work on anything other than the projects I have been assigned to” represents a reverse-coded item. To ensure alignment with the direction of other items, this item was reverse-coded before conducting the analysis. Cronbach's alpha was then calculated using the reverse-coded data, yielding a value of 0.825, which indicates high internal consistency of the scale. In contrast, without reverse-coding, the internal consistency was adversely affected, resulting in a Cronbach's alpha of −0.136.

In many studies, the gender, age, educational background, and tenure of respondents were used as control variables. Therefore, demographic variables such as gender, age, and educational background were used as control variables in the empirical analysis. Gender was encoded as 0 for males and 1 for females. Age was measured in years, and education was measured in the years in which the participant completed that stage of education.

## Results

### Descriptive statistics

Descriptive statistical analyses were performed for demographic variables based on data collected from 382 participants. Of the participants, 54.45% were males and 45.44% were females. In terms of age, participants aged 41–45 accounted for the highest proportion at 27.49%, followed by participants aged 36–40, accounting for 25.13%; the participants aged 20–25 years old had the lowest percentage at 1.31%. In terms of academic qualifications, the proportion of participants with a junior college education was the highest at 30.63%. The proportion of participants with a high school education or below was the lowest at 9.69%. In terms of tenure, 6–10 years accounted for the highest proportion (37.53). Descriptive data are shown in Table 1.

**Table 1:** Descriptive analysis of participants.

**Demographic variable**	**Type**	**Frequency**	**Ratio (%)**
Gender	Male	208	54.45
	Female	174	45.55
Age	20–25 years old	5	1.31
	26–30 years old	30	7.85
	31–35 years old	73	19.11
	36–40 years old	96	25.13
	41–45 years old	105	27.49
	46–50 years old	28	7.33
	Over 51 years old	45	11.78
Educational background	Junior high school and below	37	9.69
	High school/technical secondary school	63	16.49
	College	117	30.63
	Bachelor's degree	98	25.65
	Master's degree	67	17.54
	Doctorate	0	0
Tenure	0–5 years	92	24.15
	6–10 years	143	37.53
	11–15 years	62	16.27
	16–20 years	40	10.50
	21–25 years	19	4.99
	Over 25 years	25	6.56
Total		382	100

### Confirmatory factor analysis and reliability analysis

A confirmatory factor analysis was used to test the model fit. In this study, the fitting results of the one-factor model and the research model were compared. The fitting index showed that the research model was significantly better than the one-factor model. The results were as follows: X^2^ (*p*) = 653.138(0.000), X^2^/df = 2.677, RMSEA = 0.066, CFI = 0.923, TLI = 0.912, and SRMR = 0.057. The fitting index of the model showed a good effect. Although the chi-square test results (X^2^ = 653.138, *p* = 0.000) showed a significant difference in model fitting, the chi-square/degree-of-freedom ratio (X^2^/df = 2.677) indicated that it was acceptable, considering the effect of large samples. RMSEA values < 0.05 reflect a small approximation error, those between 0.05 and 0.08 reflect an acceptable approximation error, and values >0.10 reflect a poor model fit (Cudeck and Browne, 1992). The RMSEA value was 0.066, which was lower than 0.08, indicating that the model fit of data was good. The CFI and TLI values were 0.923 and 0.912, respectively. Both were >0.90, indicating that the model had a high degree of fit. In addition, the SRMR value was 0.057, which was < 0.08, further verifying that the model fit was good. In contrast, the fit indices for the one-factor model were poor (CFI = 0.663, TLI = 0.628, RMSEA = 0.137, SRMR = 0.103). Consequently, the research model is better able to interpret the data structure.

The average variance extraction (AVE) and composite reliability (CR) were analyzed. The AVE value measures the proportion of explanatory variance for each indicator in the construct (Hair et al., 2017), and the values for each variable is as follows: 0.721 for ethical leadership, 0.433 for psychological wellbeing, 0.541 for psychological entitlement, and 0.570 for bootlegging innovation behavior. Except for psychological wellbeing, all values were >0.5, indicating good convergent validity. The AVE value for psychological wellbeing was 0.433, which was acceptable since it was between 0.36 and 0.5 (Fornell and Larcker, 1981).

The CR value measures the internal consistency of the construct (Sen, 1993). The CR values for each variable were as follows: 0.928 for ethical leadership, 0.886 for psychological wellbeing, 0.850 for psychological entitlement, and 0.825 for bootlegging innovation behavior. All values are >0.7, indicating good confidence.

A reliability analysis was used to assess the agreement between items in a questionnaire or scale (Tavakol and Dennick, 2011). The Cronbach's alpha values calculated in this study were as follows: ethical leadership was 0.928, psychological wellbeing was 0.886, psychological entitlement was 0.850, and bootlegging innovation behavior was 0.825, all of which were >0.80. This indicated a high degree of agreement between the scale items. The results are summarized in Table 2.

**Table 2:** Confirmatory factor analysis and reliability analysis.

**Variables**	**Items**	**Estimate**	**SE**	***p***	**AVE**	**CR**	**Cronbach's alpha**
Ethical leadership	EL1				0.721	0.928	0.928
	EL2	1.028	0.046	^***^			
	EL3	1.000	0.048	^***^			
	EL4	0.964	0.047	^***^			
	EL5	0.815	0.040	^***^			
Psychological wellbeing	WB1				0.433	0.882	0.886
	WB2	1.077	0.123	^***^			
	WB3	1.300	0.138	^***^			
	WB4	1.484	0.140	^***^			
	WB5	1.045	0.127	^***^			
	WB6	1.258	0.130	^***^			
	WB7	1.149	0.113	^***^			
	WB8	1.340	0.130	^***^			
	WB9	1.250	0.120	^***^			
	WB10	1.203	0.123	^***^			
Psychological entitlement	PO1				0.541	0.854	0.850
	PO2	0.747	0.061	^***^			
	PO3	0.819	0.073	^***^			
	PO4	0.942	0.077	^***^			
	PO5	0.931	0.068	^***^			
Bootlegging innovation behavior	BIB1				0.570	0.838	0.825
	BIB2	1.479	0.140	^***^			
	BIB3	1.547	0.141	^***^			
	BIB4	1.366	0.128	^***^			
Model fit index	X^2^ (*p*) = 653.138 (0.000), X^2^/df = 2.677, RMSEA = 0.066, CFI = 0.923, TLI = 0.912, SRMR = 0.057						

^***^p < 0.001.

### Correlation analysis

The mean values for ethical leadership, psychological wellbeing, psychological entitlement, and bootlegging innovation behavior were 5.813, 5.596, 5.430, and 5.658, respectively. The standard deviations (SD) of ethical leadership, psychological wellbeing, psychological entitlement, and bootlegging innovation behavior were 1.048, 0.749, 1.149, and 0.963, respectively. Ethical leadership was associated with psychological wellbeing (*r* = 0.462, *p* < 0.001), psychological entitlement (*r* = 0.473, *p* < 0.001), and bootlegging innovation behavior (*r* = 0.519, *p* < 0.001). Psychological wellbeing was positively correlated with psychological entitlement (*r* = 0.480, *p* < 0.001) and bootlegging innovation behavior (*r* = 0.528, *p* < 0.001). Psychological entitlement was positively correlated with bootlegging innovation behavior (*r* = 0.617, *p* < 0.001). The above results are presented in Table 3.

**Table 3:** Correlation analysis.

	**Mean**	**SD**	**Gender**	**Age**	**EB**	**T**	**EL**	**WB**	**PE**	**BIB**
Gender	0.455	0.499	1							
Age	4.387	1.451	−0.154^***^	1						
EB	3.249	1.205	0.042	−0.316^***^	1					
T	2.542	1.424	−0.234^***^	0.553^***^	−0.281^***^	1				
EL	5.813	1.048	−0.032	0.036	−0.100^*^	0.011	1			
WB	5.596	0.749	−0.054	−0.065	0.154^***^	−0.039	0.462^***^	1		
PE	5.430	1.149	0.066	0.048	0.040	0.041	0.473^***^	0.480^***^	1	
BIB	5.658	0.963	0.022	−0.050	0.074	0.008	0.519^***^	0.528^***^	0.617^***^	1

^***^p < 0.001, ^*^p < 0.05.

EB, educational background; T, tenure; EL, ethical leadership; WB, psychological wellbeing; PE, psychological entitlement; BIB, bootlegging innovation behavior.

### Chained mediation analysis

The M-plus serial multiple mediation model was used to examine how the variables used in this study interacted. To test the indirect effects of the mediation and sequence mediation hypotheses, the bootstrap method was used to perform an analysis using the M-plus statistical analysis software. If the upper and lower bounds of the coefficients obtained in the middle of the 95% confidence interval (IC) do not include “0,” they can be determined as significant values (Lee et al., 2022). Table 4 lists the results of the path analysis. The path analysis results showed that ethical leadership had a direct positive effect on bootlegging innovation behavior (β = 0.228, *p* < 0.001), and Hypothesis 1 was supported. The results of the bootstrap method showed that ethical leadership → psychological wellbeing → bootlegging innovation behavior.

**Table 4:** Path analysis and bootstrap indirect effect test.

**Path**	**Estimate**	**S.E**.	***t***	***p***	**LLCI**	**ULCI**
EL → WB	0.462	0.050	9.301	0.000	0.358	0.552
EL → PE	0.314	0.060	5.217	0.000	0.190	0.427
WB → PE	0.344	0.052	6.587	0.000	0.240	0.445
WB → BIB	0.218	0.057	3.849	0.000	0.112	0.331
PE → BIB	0.403	0.061	6.609	0.000	0.283	0.520
EL → BIB	0.228	0.062	3.656	0.000	0.106	0.347
**Indirect effect**	**Effect**	**Boot SE**	**Boot LLCI**		**Boot ULCI**	
EL → WB → BIB	0.093	0.026	0.046		0.147	
EL → PE → BIB	0.116	0.033	0.060		0.191	
EL → WB → PE → BIB	0.059	0.015	0.036		0.095	
Total	0.477	0.055	0.372		0.587	

N = 382. The indirect effect estimated was tested for significance using 95% bias-corrected, bootstrapped confidence intervals. Bootstrap resampling = 5,000.

EB, educational background; T, tenure; EL, ethical leadership; WB, psychological wellbeing; PE, psychological entitlement; BIB, bootlegging innovation behavior.

There was a positive mediating effect on bootlegging innovation behavior (β = 0.093, CI: 0.046–0.147), and Hypothesis 2 was supported. The positive mediating effect of ethical leadership → psychological entitlement → bootlegging innovation behavior (β = 0.116, CI: 0.060–0.191) was also confirmed, supporting Hypothesis 3. In addition, the sequential continuous positive mediating effect of ethical leadership → psychological wellbeing → psychological entitlement → bootlegging innovation behavior (β = 0.059, CI: 0.036–0.095) did not include 0 at the 95% confidence level. Psychological wellbeing and entitlement mediate the relationship between ethical leadership and bootlegging innovation behavior. Ethical leadership adds psychological wellbeing, psychological entitlement, and psychological entitlement adds bootlegging innovation behavior, Hypothesis 4 is supported.

## Discussion

This study analyzes the behavior of organizational members from the psychological perspective, which is key to understanding and analyzing their behavior. The bootlegging innovation behavior of an organization's members is an important factor in the change and development of an enterprise. Therefore, from the perspective of employee psychology, this study analyzes the relationship between ethical leadership and employees' bootlegging innovation behavior as well as the mediating role of psychological wellbeing and psychological entitlement. The empirical analysis supports our hypothesis that ethical leadership has a significant direct impact on bootlegging innovation behavior, and psychological wellbeing and psychological entitlement are two antecedents that have an important impact on bootlegging innovation behavior. The results show that both psychological wellbeing and entitlement mediate the relationship between ethical leadership and employees' bootlegging innovation behavior. Additionally, this study found a chain mediation effect. In other words, ethical leadership not only has a direct positive impact on employees' bootlegging innovation behavior but also indirectly affects employees' bootlegging innovation behavior by increasing their psychological wellbeing and psychological entitlement. Ethical leadership often leaves an impression of reliability and integrity in the minds of employees. Employees feel encouraged and affirmed by the leader, which in turn increases their psychological wellbeing, encourages them to have a positive attitude toward their work and organization, and believes that their efforts are worthwhile. Subsequently, they tend to perceive their ability to work and thus want to gain a higher status and more opportunities to prove their abilities, which increases their psychological entitlement. In addition, employees in this state are highly willing to actively participate in the organization's activities and expect to contribute to the organization by repaying their leaders' care. As a result, employees often do not worry about being penalized for unauthorized innovation, or that employees have a belief in successful completion; they believe they have ability to complete the innovation and bring benefits to the leadership and the organization and will not be penalized for it, which leads to an increase in employee bootlegging innovation behavior.

This research provides an explanatory possibility for exploring the relationship between ethical leadership, psychological wellbeing, psychological entitlement, and bootlegging innovation behavior also provides a new perspective for the study of bootlegging innovation behavior.

## Implications

### Theoretical contribution

This study expands on the existing research results on leadership and employee innovation behavior and first hypothesizes and verifies the mediating process of the relationship between employees' psychological wellbeing and psychological entitlement to ethical leadership and employees' bootlegging innovation behavior. Much of the past research on bootlegging innovation behavior has focused on individual characteristics and organizational factors (Globocnik and Salomo, 2015; Ghasemzadeh et al., 2021), while the individual psychological factors that promote bootlegging innovation behavior among employees are relatively understudied (Krueger and Buchwald, 2019). This study explores the explanatory mechanism of the impact of ethical leadership on employees' bootlegging innovation behavior from a psychological perspective and expands the research on leadership and employee creativity. The results show that psychological wellbeing wellbeing and psychological entitlement constitute the influences of ethical leadership on bootlegging innovation This study identifies the leadership factors and methods for ethical leadership that promote employees' bootlegging innovation behavior.

Previous studies have explored the effects of different leadership styles on bootlegging innovation behavior, such as the impact of abusive supervision (Wang H. et al., 2023) and agile leadership (Hooi and Tan, 2021) on bootlegging behavior, focusing primarily on direct impacts. We used a sequence mediation model and found a sequence double mediating effect in the relationship between ethical leadership and bootlegging innovation behavior. Previous studies have not included the positive effects of psychological wellbeing and psychological entitlement on bootlegging innovation behaviors influenced by ethical leadership. In addition, by examining the dual mediating effect, this study found and confirmed the individual mediating effect of psychological wellbeing and psychological entitlement, as well as a series of interrelated mediating effects, which will be beneficial in expanding the current research on leadership and employee innovation behavior.

Based on the social learning theory (Pandura, 1977), this study expands its applicability of social learning theory in the field of bootlegging behavior by demonstrating that ethical leadership affects psychological wellbeing, psychological rights, and bootlegging innovation behavior.

### Practical contribution

Bootlegging innovation behavior is risky; however, in many cases, it can lead to unexpected innovation. Recent studies have pointed to the tendency of bootlegging innovation behavior to become a common practice in organizations and the positive impact of this tendency on organizational innovation (Globocnik et al., 2022). Therefore, by implementing ethical leadership, enterprises can stimulate employees' innovation potential without compromising organizational rules and ethical principles, thereby improving their overall innovation ability and market competitiveness. The positive effects of ethical leadership include a harmonious and friendly working environment and a respectful and caring relationship among members of the organization. Therefore, the organization should promote and maintain a positive and agreeable organizational culture so that if the organization's members make innovative suggestions that have not been adopted, they may bring additional benefits and results.

Second, psychological wellbeing positively mediates the relationship between ethical leadership and bootlegging innovation behavior. In other words, enterprises can promote the psychological wellbeing of employees by promoting ethical leadership, which will help increase employees' willingness and enthusiasm to participate in innovation activities and is also of great significance for improving employees' job satisfaction and overall wellbeing.

Psychological entitlement also positively mediates the relationship between ethical leadership and bootlegging innovation behavior. Understanding the relationships among ethical leadership, psychological wellbeing, psychological entitlement, and bootlegging innovation behavior can help managers design and implement better management practices. In addition, the results of this study can guide leadership training courses for businesses and organizations. This study shows that organizations can improve the leadership quality of managers by strengthening ethical leadership training, improving employees' psychological wellbeing and psychological entitlement, and stimulating innovative behaviors.

## Limitations and future research

This study helps validate the role of psychological wellbeing and psychological entitlement in the relationship between ethical leadership and bootlegging innovation behavior. However, it also has some limitations, as discussed below. The samples in this study were mainly from specific industries or regions, which may have affected the generalizability of the research results. Factors such as cultural background, industry characteristics, and firm size may affect the relationships in ethical leadership, psychological wellbeing, psychological entitlement, and bootlegging innovation behavior. Therefore, future research should be conducted in different countries, cultural backgrounds, and types of enterprises to verify the universality of the conclusions.

Furthermore, this study mainly used a questionnaire survey method to collect data; although this method can obtain a large amount of data, it has some limitations. Since the data were collected from the same source at the same time, there may be a problem with common method variance. Future research should consider the possibility of a reverse causal relationship. Future research could adopt a combination of multiple research methods such as experimental research, longitudinal research, and case studies to overcome the limitations of a single method. For example, experimental methods and longitudinal study designs have been used to better reveal the dynamic processes between variables.

It is important to note that whether bootlegging behavior leads to innovation or result in a waste of organizational time and resources largely depends on the working environment in which these activities occur (Criscuolo et al., 2014). The willingness to autonomously develop and innovate ideas requires a certain degree of accountability to ensure balance (Kanter, 2009). If this freedom is not properly managed and individual innovation efforts no longer align with the needs and goals of the organization (Criscuolo et al., 2014), it may lead to undesirable outcomes, such as management chaos and decreased efficiency. In extreme cases, the work environment may prioritize strict adherence to norms and rules (Mainemelis, 2010). For instance, pressure to achieve formal goals in underperforming organizations may compel local managers to enforce adherence to organizational norms (Criscuolo et al., 2014). In such contexts, bootlegging may be seen as contributing to resource waste, particularly in organizations with a poor reputation for innovation. Research by Criscuolo et al. (2014) suggests that the positive effects of bootlegging on individual innovation performance are weaker in organizations with low levels of bootlegging, as these units have limited experience in overcoming the barriers to adopting the concept of “breaking out of apathy.” Therefore, whether bootlegging benefits the organization depends on the work environment, and the decision to encourage bootlegging should be made in the context of the organization's specific management practices.

Finally, this study examines the relationships between ethical leadership, psychological wellbeing, psychological entitlement, and bootlegging innovation behavior. Future research should introduce additional mediating and moderating variables to construct a more comprehensive theoretical model. For example, factors such as organizational culture, team atmosphere, and employee personality traits may play important roles in these relationships.

As digital transformation advances, the ways in which organizations manage and evolve, and how employees work, have changed dramatically. Future research can explore how ethical leadership affects employees' bootlegging innovation behaviors in the context of digitalization, especially digital bootlegging innovation behaviors, and analyze the impact of digital environment on the effectiveness of ethical leadership.

## Data Availability

The original contributions presented in the study are included in the article/supplementary material, further inquiries can be directed to the corresponding authors.

## References

[B1] Abiodun R. A. (2010). Leadership Behavior Impact on Employee's Loyalty, Engagement and Organizational Performance: Leadership Behavior and Employee Perception of the Organization. Bloomington: AuthorHouse.

[B2] Aggarwal-Gupta M. Vohra N. Bhatnagar D. (2010). Perceived organizational support and organizational commitment: the mediational influence of psychological well-being. J. Bus. Manag. 16, 105–124. 10.1504/JBM.2010.14118235009967

[B3] Ahmed Iqbal Z. Abid G. Contreras F. Hassan Q. Zafar R. (2020). Ethical leadership and innovative work behavior: the mediating role of individual attributes. J. Open Innov. Technol. Mark. Complex. 6:68. 10.3390/joitmc6030068

[B4] Amore M. D. Garofalo O. Guerra A. (2023). How leaders influence (un) ethical behaviors within organizations: a laboratory experiment on reporting choices. J. Bus. Ethics 183, 495–510. 10.1007/s10551-022-05088-z

[B5] Ampofo E. T. (2021). Do job satisfaction and work engagement mediate the effects of psychological contract breach and abusive supervision on hotel employees' life satisfaction? J. Hosp. Mark. Manag. 30, 282–304. 10.1080/19368623.2020.1817222

[B6] Antoncic B. (2007). Intrapreneurship: a comparative structural equation modeling study. Indust. Manag. Data Syst. 107, 309–325. 10.1108/02635570710734244

[B7] Aronson E. (2001). Integrating leadership styles and ethical perspectives. Can. J. Admin. Sci. 18, 244–256. 10.1111/j.1936-4490.2001.tb00260.x35581581

[B8] Augsdorfer P. (2005). Bootlegging and path dependency. Res. Policy 34, 1–11. 10.1016/j.respol.2004.09.010

[B9] Avolio B. J. Walumbwa F. O. Weber T. J. (2009). Leadership: current theories, research, and future directions. Annu. Rev. Psychol. 60, 421–449. 10.1146/annurev.psych.60.110707.163621 18651820

[B10] Bahmannia S. van Knippenberg D. Lowe K. B. (2023). Even nectar is poisonous in excess: the impact of leader humility on pride, entitlement, and organizational citizenship behavior. J. Lead. Organ. Stud. 30, 381–396. 10.1177/15480518231204675

[B11] Bedi A. Alpaslan C. M. Green S. (2016). A meta-analytic review of ethical leadership outcomes and moderators. J. Bus. Ethics 139, 517–536. 10.1007/s10551-015-2625-136619057

[B12] Bello S. M. (2012). Impact of ethical leadership on employee job performance. Int. J. Bus. Soc. Sci. 3, 228–236.

[B13] Bennett R. J. Galperin B. L. Wang L. Shukla J. (2024). Norm-violating behavior in organizations: a comprehensive conceptual review and model of constructive and destructive norm-violating behavior. Annu. Rev. Organ. Psychol. Organ. Behav. 11, 481–507. 10.1146/annurev-orgpsych-110721-043001

[B14] Berraies S. Lajili R. Chtioui R. (2020). Social capital, employees' well-being and knowledge sharing: does enterprise social networks use matter? Case of Tunisian knowledge-intensive firms. J. Intellect. Capital 21, 1153–1183. 10.1108/JIC-01-2020-0012

[B15] Black H. C. Garner B. A. McDaniel B. R. Schultz D. W. (1999). Black's Law Dictionary. Eagan: West Publishing Company. 196.

[B16] Blanchflower D. G. Oswald A. J. (2008). Is well-being U-shaped over the life cycle?. Soc. Sci. Med. 66, 1733–1749. 10.1016/j.socscimed.2008.01.030 18316146

[B17] Bless H. Mackie D. M. Schwarz N. (1992). Mood effects on attitude judgments: independent effects of mood before and after message elaboration. J. Pers. Soc. Psychol. 63:585. 10.1037/0022-3514.63.4.585 1447687

[B18] Bradley W. A. Kolev J. (2023). How does digital piracy affect innovation? Evidence from software firms. Res. Policy 52:104701. 10.1016/j.respol.2022.104701

[B19] Brown M. E. Treviño L. K. (2006). Ethical leadership: a review and future directions. Leadersh. Q. 17, 595–616. 10.1016/j.leaqua.2006.10.004

[B20] Brown M. E. Treviño L. K. Harrison D. A. (2005). Ethical leadership: a social learning perspective for construct development and testing. Organ. Behav. Hum. Decis. Process. 97, 117–134. 10.1016/j.obhdp.2005.03.00239678304

[B21] Campbell W. K. Bonacci A. M. Shelton J. Exline J. J. Bushman B. J. (2004). Psychological entitlement: interpersonal consequences and validation of a self-report measure. J. Pers. Assess. 83, 29–45. 10.1207/s15327752jpa8301_04 15271594

[B22] Canter D. (Ed.). (2012). Facet Theory: Approaches to Social Research. Springer Science and Business Media.

[B23] Charles-Leija H. Castro C. G. Toledo M. Ballesteros-Valdés R. (2023). Meaningful work, happiness at work, and turnover intentions. Int. J. Environ. Res. Public Health 20:3565. 10.3390/ijerph20043565 36834260 PMC9963286

[B24] Chen M. Zheng X. Wu B. (2024). Why is leader humility related to OCBs? A psychological entitlement explanation of the curvilinear moderated relations. Leadersh. Organ. Dev. J. 45, 1028–1047. 10.1108/LODJ-06-2023-0332

[B25] Chevalier A. Feinstein L. (2006). Sheepskin or Prozac: the causal effect of education on mental health. SSRN Electr. J. 1–45. 10.2139/ssrn.923530

[B26] Ciulla J. B. (2004). Ethics and leadership effectiveness. Nat. Leadersh. 11, 302–327. 10.5040/9798216967286

[B27] Cook K. S. Emerson R. M. (1987). Social Exchange Theory. Newbury Park: Taylor & Francis.

[B28] Costa P. T. McCrae R. R. (1980). Influence of extraversion and neuroticism on subjective well-being: happy and unhappy people. J. Pers. Soc. Psychol. 38:668. 10.1037/0022-3514.38.4.668 7381680

[B29] Covington B. (2023). Empower Your Nursing Leadership: A Comprehensive Guide to Career Advancement and Positive Work Environments. BIngramSpark,

[B30] Crampton S. M. Hodge J. W. (2009). Generation Y: unchartered territory. J. Bus. Econ. Res. 7, 1–6. 10.19030/jber.v7i4.2272

[B31] Criscuolo P. Salter A. Ter Wal A. L. (2014). Going underground: bootlegging and individual innovative performance. Organ. Sci. 25, 1287–1305. 10.1287/orsc.2013.085619642375

[B32] Cropanzano R. Mitchell M. S. (2005). Social exchange theory: an interdisciplinary review. J. Manage. 31, 874–900. 10.1177/0149206305279602

[B33] Cudeck R. Browne M. W. (1992). Constructing a covariance matrix that yields a specified minimizer and a specified minimum discrepancy function value. Psychometrika 57, 357–369. 10.1007/BF02295424

[B34] De Clercq D. (2023). The damage of deference: how personal and organizational factors transform deference to leader authority into unethical pro-organizational behavior. Manag. Res. Rev. 46, 1637–1660. 10.1108/MRR-08-2022-0602

[B35] De Jong J. P. Den Hartog D. N. (2007). How leaders influence employees' innovative behaviour. Eur. J. Innov. Manag. 10, 41–64. 10.1108/14601060710720546

[B36] De Roeck K. Farooq O. (2018). Corporate social responsibility and ethical leadership: investigating their interactive effect on employees' socially responsible behaviors. J. Bus. Ethics 151, 923–939. 10.1007/s10551-017-3656-634566768

[B37] Den Hartog D. N. (2015). Ethical leadership. Annu. Rev. Organ. Psychol. Organ. Behav. 2, 409–434. 10.1146/annurev-orgpsych-032414-111237

[B38] Den Hartog D. N. De Hoogh A. H. (2009). Empowering behaviour and leader fairness and integrity: studying perceptions of ethical leader behaviour from a levels-of-analysis perspective. Eur. J. Work Organ. Psychol. 18, 199–230. 10.1080/13594320802362688

[B39] Durán-Sandoval D. Uleri F. (2023). Definition of economics in retrospective: two epistemological tensions that explain the change of the study object in economics. Philosophies 9:1. 10.3390/philosophies9010001

[B40] Erkutlu H. Chafra J. (2016). Benevolent leadership and psychological well-being: the moderating effects of psychological safety and psychological contract breach. Leadersh. Organ. Dev. J. 37, 369–386. 10.1108/LODJ-07-2014-0129

[B41] Evans J. Repper J. (2000). Employment, social inclusion and mental health. J. Psychiatr. Ment. Health Nurs. 7, 15–24. 10.1046/j.1365-2850.2000.00260.x 11022507

[B42] Fagg J. Curtis S. Stansfeld S. A. Cattell V. Tupuola A. M. Arephin M. (2008). Area social fragmentation, social support for individuals and psychosocial health in young adults: evidence from a national survey in England. Soc. Sci. Med. 66, 242–254. 10.1016/j.socscimed.2007.07.032 17988774

[B43] Feather N. T. (1999). Judgments of deservingness: studies in the psychology of justice and achievement. Pers. Soc. Psychol. Rev. 3, 86–107. 10.1207/s15327957pspr0302_1 15647142

[B44] Fornell C. Larcker D. F. (1981). Evaluating structural equation models with unobservable variables and measurement error. J. Mark. Res. 18, 39–50. 10.1177/002224378101800104

[B45] Fredrickson B. L. Branigan C. (2005). Positive emotions broaden the scope of attention and thought-action repertoires. Cogn. Emot. 19, 313–332. 10.1080/02699930441000238 21852891 PMC3156609

[B46] Fredrickson B. L. Joiner T. (2002). Positive emotions trigger upward spirals toward emotional well-being. Psychol. Sci. 13, 172–175. 10.1111/1467-9280.00431 11934003

[B47] Galvin B. M. Lange D. Ashforth B. E. (2015). Narcissistic organizational identification: seeing oneself as central to the organization's identity. Acad. Manag. Rev. 40, 163–181. 10.5465/amr.2013.0103

[B48] Gao R. Liu B. (2023). Avoiding the scenario of “The farmer and the snake”: the dark side of servant leadership and an intervention mechanism. J. Manag. Psychol. 38, 289–302. 10.1108/JMP-02-2022-0062

[B49] García-Sánchez E. Matamoros-Lima J. Moreno-Bella E. Melita D. Sánchez-Rodríguez Á. García-Castro J. D.. (2024). Perceived economic inequality is negatively associated with subjective well-being through status anxiety and social trust. Soc. Indic. Res. 172, 239–260. 10.1007/s11205-024-03306-x

[B50] Gasper K. Clore G. L. (2000). Do you have to pay attention to your feelings to be influenced by them?. Pers. Soc. Psychol. Bull. 26, 698–711. 10.1177/0146167200268005

[B51] Ghasemzadeh K. Bunjak A. Bortoluzzi G. Cerne M. (2021). Efficaciously smuggling ideas: untangling the relationship between entrepreneurial self-efficacy, creative bootlegging and embedded lead users. Int. J. Innov. Manag. 25, 1–23. 10.1142/S1363919621500328

[B52] Globocnik D. (2019). Taking or avoiding risk through secret innovation activities-the relationships among employees‘ risk propensity, bootlegging, and management support. Int. J. Innov. Manag. 23, 1–41. 10.1142/S1363919619500221

[B53] Globocnik D. (2023). “Individual and contextual factors affecting employees' inclination to bootlegging,” in Corporate Underground: Bootleg Innovation and Constructive Deviance, ed. P. Augsdorfer (Singapore: World Scientific), 167–186.

[B54] Globocnik D. Peña Häufler B. Salomo S. (2022). Organizational antecedents to bootlegging and consequences for the newness of the innovation portfolio. J. Prod. Innov. Manag. 39, 717–745. 10.1111/jpim.12626

[B55] Globocnik D. Salomo S. (2015). Do formal management practices impact the emergence of bootlegging behavior? J. Prod. Innov. Manag. 32, 505–521. 10.1111/jpim.12215

[B56] Goldsby M. G. Kuratko D. F. Hornsby J. S. Houghton J. D. Neck C. P. (2006). Social cognition and corporate entrepreneurship: a framework for enhancing the role of middle-level managers. Int. J. Leadersh. Stud. 2, 17–35.

[B57] Grant R. M. (2003). Strategic planning in a turbulent environment: evidence from the oil majors. Strateg. Manag. J. 24, 491–517. 10.1002/smj.314

[B58] Guberina T. Wang A. M. Obrenovic B. (2023). An empirical study of entrepreneurial leadership and fear of COVID-19 impact on psychological wellbeing: a mediating effect of job insecurity. PLoS ONE 18:e0284766. 10.1371/journal.pone.0284766 37172060 PMC10180687

[B59] Hair J. F. Jr. Matthews L. M. Matthews R. L. Sarstedt M. (2017). PLS-SEM or CB-SEM: updated guidelines on which method to use. Int. J. Multivar. Data Anal. 1, 107–123. 10.1504/IJMDA.2017.08762435009967

[B60] Harvey P. Harris K. J. (2010). Frustration-based outcomes of entitlement and the influence of supervisor communication. Hum. Relat. 63, 1639–1660. 10.1177/0018726710362923

[B61] Harvey P. Martinko M. J. (2009). An empirical examination of the role of attributions in psychological entitlement and its outcomes. J. Organ. Behav. 30, 459–476. 10.1002/job.549

[B62] He P. X. Wu T. J. Zhao H. D. Yang Y. (2019). How to motivate employees for sustained innovation behavior in job stressors? A cross-level analysis of organizational innovation climate. Int. J. Environ. Res. Public Health 16:4608. 10.3390/ijerph16234608 31757069 PMC6926950

[B63] Helliwell J. F. (2003). How's life? Combining individual and national variables to explain subjective well-being. Econ. Model. 20, 331–360. 10.1016/S0264-9993(02)00057-3

[B64] Helzer E. G. Kim S. H. (2019). Creativity for workplace well-being. Acad. Manag. Perspect. 33, 134–147. 10.5465/amp.2016.0141

[B65] Hoang G. Luu T. T. Du T. Nguyen T. T. (2023). Can both entrepreneurial and ethical leadership shape employees' service innovative behavior?. J. Serv. Market. 37, 446–463. 10.1108/JSM-07-2021-0276

[B66] Hooi L. W. Tan N. N. (2021). “Agile leadership and bootlegging behavior: does leadership coping dynamics matter?,” in Agile Coping in the Digital Workplace: Emerging Issues for Research and Practice, eds. N. Ferreira, I. L. Potgieter, and M. Coetzee (Cham: Springer International Publishing), 187–202.

[B67] Hougaard R. Carter J. (2018). The Mind of the Leader: How to Lead Yourself, Your People, and Your Organization for Extraordinary Results. Brighton, MA: Harvard Business Press.

[B68] Howell J. M. (2005). The right stuff: identifying and developing effective champions of innovation. Acad. Manag. Perspect. 19, 108–119. 10.5465/ame.2005.16965104

[B69] Howell J. M. Higgins C. A. (1990). Champions of technological innovation. Administr. Sci. Q. 35, 317–341. 10.2307/2393393

[B70] Huang D. Zhu T. Wu Y. Sun T. (2022). A study on paradoxical leadership and multiple path mechanisms of employees' Bootleg innovation. Psychol. Res. Behav. Manag. 15, 3391–3407. 10.2147/PRBM.S383155 36444276 PMC9700461

[B71] Huppert F. A. (2009). Psychological well-being: evidence regarding its causes and consequences. Appl. Psychol. Health Well Being 1, 137–164. 10.1111/j.1758-0854.2009.01008.x

[B72] Ilyas S. Abid G. Ashfaq F. (2023). Enhancing the perceived organizational support, perceived ethical-philanthropic CSR and subjective well-being: the role of ethical leadership. Int. J. Ethics Syst. 39, 713–736. 10.1108/IJOES-04-2022-0084

[B73] Jensen L. (2023). Beyond rank attainment: examining the nature and function of dominance and prestige in teams (Electronic Thesis and Dissertation Repository), 9780. Available at: https://ir.lib.uwo.ca/etd/9780

[B74] Jiang W. Liang B. Wang L. (2023). The double-edged sword effect of unethical pro-organizational behavior: the relationship between unethical pro-organizational behavior, organizational citizenship behavior, and work effort. J. Bus. Ethics 183, 1159–1172. 10.1007/s10551-021-05034-5

[B75] Kalshoven K. Den Hartog D. N. De Hoogh A. H. (2011). Ethical leadership at work questionnaire (ELW): development and validation of a multidimensional measure. Leadersh. Q. 22, 51–69. 10.1016/j.leaqua.2010.12.007

[B76] Kalshoven K. Den Hartog D. N. de Hoogh A. H. (2013). Ethical leadership and followers' helping and initiative: the role of demonstrated responsibility and job autonomy. Eur. J. Work Organ. Psychol. 22, 165–181. 10.1080/1359432X.2011.640773

[B77] Kanter R. M. (2006). Innovation: the classic traps. Harv. Bus. Rev. 84, 72–83. 17131564

[B78] Kanter R. M. (2009). “When a thousand flowers bloom: structural, collective, and social conditions for innovation in organizations,” in Knowledge Management and Organisational Design (Routledge), 93–131.

[B79] Khan S. R. Javed U. (2018). Revision of ethical leadership scale. J. Res. Reflect. Educ. 12, 121–135. 10.1177/1942775117718110

[B80] Kim A. J. Chung M. H. (2023). Psychological ownership and ambivalent employee behaviors: a moderated mediation model. SAGE Open 13, 1–19. 10.1177/21582440231162535

[B81] King R. B. Cai Y. Elliot A. J. (2024). Income inequality is associated with heightened test anxiety and lower academic achievement: a cross-national study in 51 countries. Learn. Instruct. 89:101825. 10.1016/j.learninstruc.2023.101825

[B82] Koch R. Leitner K. H. (2008). The dynamics and functions of self-organization in the fuzzy front end: empirical evidence from the Austrian semiconductor industry. Creativ. Innov. Manag. 17, 216–226. 10.1111/j.1467-8691.2008.00488.x

[B83] Krivenko E. Y. (2023). “The traditional logic of human rights and the subject/object dichotomy,” in The Logic of Human Rights (Cheltenham: Edward Elgar Publishing), 35–76.

[B84] Krueger A. Buchwald A. (2019). “Motivation as determinant of bootlegging innovation,” in The ISPIM Innovation Conference (Florence), 1–10.

[B85] Krueger A. Buchwald A. (2023). “The individual in bootlegging innovation,” in Corporate Underground: Bootleg Innovation and Constructive Deviance, ed. P. Augsdorfer (Singapore: World Scientific), 279–290.

[B86] Krueger N. F. Jr. Reilly M. D. Carsrud A. L. (2000). Competing models of entrepreneurial intentions. J. Bus. Ventur. 15, 411–432. 10.1016/S0883-9026(98)00033-0

[B87] Kurth M. (2023). Use or lose: the subtle effect of corporate social responsibility on workers' psychological capital. Acad. Manag. 1:10668. 10.5465/AMPROC.2023.24bp

[B88] Lange J. Redford L. Crusius J. (2019). A status-seeking account of psychological entitlement. Pers. Soc. Psychol. Bull. 45, 1113–1128. 10.1177/0146167218808501 30486751 PMC6552293

[B89] Lee A. Schwarz G. Newman A. Legood A. (2019). Investigating when and why psychological entitlement predicts unethical pro-organizational behavior. J. Bus. Ethics 154, 109–126. 10.1007/s10551-017-3456-z

[B90] Lee W. R. Kang S. W. Choi S. B. (2022). Abusive supervision and employee's creative performance: a serial mediation model of relational conflict and employee silence. Behav. Sci. 12:156. 10.3390/bs12050156 35621453 PMC9137777

[B91] Lemoine G. J. Hartnell C. A. Leroy H. (2019). Taking stock of moral approaches to leadership: an integrative review of ethical, authentic, and servant leadership. Acad. Manag. Ann. 13, 148–187. 10.5465/annals.2016.0121

[B92] Lennick D. Kiel F. (2011). Moral Intelligence 2.0: Enhancing Business Performance and Leadership Success in Turbulent Times. Upper Saddle River, NJ: Pearson Prentice Hall.

[B93] Levine D. P. (2005). The corrupt organization. Hum. Relat. 58, 723–740. 10.1177/0018726705057160

[B94] Li L. Huang G. Yan Y. (2022). Coaching leadership and employees' deviant innovation behavior: mediation and chain mediation of interactional justice and organizational identification. Psychol. Res. Behav. Manag. 15, 3861–3874. 10.2147/PRBM.S381968 36597445 PMC9805742

[B95] Li M. Ye H. (2021). Temporal leadership and bootlegging behavior of employees: the mediating effect of self-efficacy. Front. Psychol. 12:633261. 10.3389/fpsyg.2021.633261 34646185 PMC8503315

[B96] Li S. Jia R. Seufert J. H. Tang H. Luo J. (2021). As the tree is, so is the fruit? Examining the effects of ethical leadership on bootlegging from the perspective of leader–follower gender similarity. Gender Manag. Int. J. 36, 785–800. 10.1108/GM-06-2020-0180

[B97] Lin B. Mainemelis C. Kark R. (2016). Leaders' responses to creative deviance: differential effects on subsequent creative deviance and creative performance. Leadersh. Q. 27, 537–556. 10.1016/j.leaqua.2015.09.001

[B98] Lin S. Y. Chen H. C. Chen I. H. (2023). The bright side of entitlement: exploring the positive effects of psychological entitlement on job involvement. Evid. HRM Glob. Forum Empirical Scholarsh. 11, 19–13. 10.1108/EBHRM-05-2021-0097

[B99] Liu X. Huang Y. Kim J. Na S. (2023). How ethical leadership cultivates innovative work behaviors in employees? Psychological safety, work engagement and openness to experience. Sustainability 15:3452. 10.3390/su15043452

[B100] Lucas R. E. Clark A. E. Georgellis Y. Diener E. (2004). Unemployment alters the set point for life satisfaction. Psychol. Sci. 15, 8–13. 10.1111/j.0963-7214.2004.01501002.x 14717825

[B101] Luthans F. (2002). Positive organizational behavior: developing and managing psychological strengths. Acad. Manag. Perspect. 16, 57–72. 10.5465/ame.2002.6640181

[B102] Lyu L. Zhang H. Gao K. (2022). Why does distributed leadership foster or hamper bootlegging behavior of employees: the role of exploratory-exploitative learning tension and paradox mindset. Mathem. Prob. Eng. 2022:3093641. 10.1155/2022/3093641

[B103] Lyubomirsky S. King L. Diener E. (2005). The benefits of frequent positive affect: does happiness lead to success? Psychol. Bull. 131:803. 10.1037/0033-2909.131.6.803 16351326

[B104] Mainemelis C. (2010). Stealing fire: creative deviance in the evolution of new ideas. Acad. Manag. Rev. 35, 558–578. 10.5465/AMR.2010.53502801

[B105] Mainemelis C. Sakellariou E. (2023). Creativity and the arts of disguise: switching between formal and informal channels in the evolution of creative projects. Organ. Sci. 34, 380–403. 10.1287/orsc.2022.157719642375

[B106] Maner J. K. Case C. R. (2016). Dominance and prestige: dual strategies for navigating social hierarchies. Adv. Exp. Soc. Psychol. 54, 129–180. 10.1016/bs.aesp.2016.02.001

[B107] Maner J. K. Hasty C. R. (2023). Life history strategies, prestige, and dominance: an evolutionary developmental view of social hierarchy. Pers. Soc. Psychol. Bull. 49, 627–641. 10.1177/01461672221078667 35227124

[B108] Mao H. Peng S. Zhang L. Zhang Y. (2023). Self-serving leadership and innovative behavior: roles of psychological entitlement and moral identity. Front. Psychol. 14:1071457. 10.3389/fpsyg.2023.1071457 36910833 PMC9995759

[B109] March J. G. (1991). How decisions happen in organizations. Hum. Comput. Interact. 6, 95–117.

[B110] Maslow A. H. (1987). Motivation and Personality. Hoboken, NJ: Pearson Education India.

[B111] Masoudnia Y. Szwejczewski M. (2012). Bootlegging in the RandD departments of high-technology firms. Res. Technol. Manag. 55, 35–42. 10.5437/08956308X5505070

[B112] Masterson S. S. Lewis K. Goldman B. M. Taylor M. S. (2000). Integrating justice and social exchange: the differing effects of fair procedures and treatment on work relationships. Acad. Manag. J. 43, 738–748. 10.2307/1556364

[B113] Morrison E. W. Phelps C. C. (1999). Taking charge at work: extrarole efforts to initiate workplace change. Acad. Manag. J. 42, 403–419. 10.2307/257011

[B114] Muñoz-Pascual L. Galende J. (2020). Ambidextrous relationships and social capability as employee well-being: the secret sauce for research and development and sustainable innovation performance. Int. J. Environ. Res. Public Health 17:3072. 10.3390/ijerph17093072 32354118 PMC7246496

[B115] Naumann S. E. Minsky B. D. Sturman M. C. (2002). The use of the concept “entitlement” in management literature: a historical review, synthesis, and discussion of compensation policy implications. Hum. Resour. Manag. Rev. 12, 145–166. 10.1016/S1053-4822(01)00055-9

[B116] Neves P. Story J. (2015). Ethical leadership and reputation: combined indirect effects on organizational deviance. J. Bus. Ethics 127, 165–176. 10.1007/s10551-013-1997-3

[B117] Neville L. Fisk G. M. Ens K. (2024). Psychological entitlement and conspiracy beliefs: evidence from the COVID-19 pandemic. J. Soc. Psychol. 165, 65–87. 10.1080/00224545.2023.2292626 38163924

[B118] Nozick R. Nagel T. (1974). Anarchy, State, and Utopia, Vol. 5038. New York, NY: Basic books.

[B119] Pandura A. (1977). Social Learning Theory.

[B120] Pennington K. (2023). The Prince and the Law. 1200-1600: Sovereignty and Rights in the Western Legal Tradition. Oakland, CA: University of California Press.

[B121] Piecek E. J. (2023). Give and you may receive: examining transparent leadership through the lens of leader-follower relationships (Thesis).

[B122] Pierce J. L. Gardner D. G. (2004). Self-esteem within the work and organizational context: a review of the organization-based self-esteem literature. J. Manage. 30, 591–622. 10.1016/j.jm.2003.10.00137213371

[B123] Qu J. Khapova S. N. Xu S. Cai W. Zhang Y. Zhang L.. (2023). Does leader humility foster employee bootlegging? Examining the mediating role of relational energy and the moderating role of work unit structure. J. Bus. Psychol. 38, 1287–1305. 10.1007/s10869-023-09884-w 37359079 PMC10149628

[B124] Reave L. (2005). Spiritual values and practices related to leadership effectiveness. Leadersh. Q. 16, 655–687. 10.1016/j.leaqua.2005.07.003

[B125] Ren S. Chadee D. (2017). Ethical leadership, self-efficacy and job satisfaction in China: the moderating role of guanxi. Pers. Rev. 46, 371–388. 10.1108/PR-08-2015-0226

[B126] Ridgeway C. L. (2014). Why status matters for inequality. Am. Sociol. Rev. 79, 1–16. 10.1177/0003122413515997

[B127] Roberts-Cady S. (2024). Rawls and economic liberties. Res Publica 1–21. 10.1007/s11158-024-09668-w

[B128] Robinson S. L. Rousseau D. M. (1994). Violating the psychological contract: not the exception but the norm. J. Organ. Behav. 15, 245–259. 10.1002/job.4030150306

[B129] Rodwell J. Ellershaw J. (2024). Suggesting context differences influence the impact of nurses' psychological contracts. Soc. Sci. 13:40. 10.3390/socsci13010040

[B130] Rose K. C. Anastasio P. A. (2014). Entitlement is about ‘others', narcissism is not: relations to sociotropic and autonomous interpersonal styles. Pers. Individ. Dif. 59, 50–53. 10.1016/j.paid.2013.11.004

[B131] Ryan R. M. Deci E. L. (2001). On happiness and human potentials: a review of research on hedonic and eudaimonic well-being. Annu. Rev. Psychol. 52, 141–166. 10.1146/annurev.psych.52.1.141 11148302

[B132] Ryff C. Singer B. (2002). “From social structure to biology,” in Handbook of Positive Psychology, eds. C. R. Snyder and S. J. Lopez (Oxford: Oxford University Press), 73.

[B133] Ryff C. D. Keyes C. L. M. (1995). The structure of psychological well-being revisited. J. Pers. Soc. Psychol. 69:719. 10.1037/0022-3514.69.4.719 7473027

[B134] Ryff C. D. Singer B. (1998). The contours of positive human health. Psychol. Inq. 9, 1–28. 10.1207/s15327965pli0901_1

[B135] Schuckert M. Kim T. T. Paek S. Lee G. (2018). Motivate to innovate: how authentic and transformational leaders influence employees' psychological capital and service innovation behavior. Int. J. Contemp. Hosp. Manag. 30, 776–796. 10.1108/IJCHM-05-2016-0282

[B136] Schwarz G. Newman A. Yu J. Michaels V. (2023). Psychological entitlement and organizational citizenship behaviors: the roles of employee involvement climate and affective organizational commitment. Int. J. Hum. Resour. Manag. 34, 197–222. 10.1080/09585192.2021.1962388

[B137] Sen A. (1993). Internal consistency of choice. Econometrica 61, 495–521. 10.2307/2951715

[B138] Shang H. (2024). Review of employees' bootleg innovation behavior. Highlights Bus. Econ. Manag. 32, 170–178. 10.54097/ghb95e54

[B139] Sheldon K. M. Lyubomirsky S. (2006). Achieving sustainable gains in happiness: change your actions, not your circumstances. J. Happiness Stud. 7, 55–86. 10.1007/s10902-005-0868-8

[B140] Shiundu T. W. (2024). Ethical leadership and its implication on decision-making in organizations: a literature review. J. Hum. Resour. Leadersh. 8, 59–67. 10.53819/81018102t30131

[B141] Si W. Shi S. Zhou M. Cai Z. (2023). Taken for granted: when servant leadership may be negatively related to OCB via psychological entitlement. J. Bus. Res. 166:114122. 10.1016/j.jbusres.2023.114122

[B142] Si X. Xue H. Song X. Liu X. Zhang F. (2023). The relationship between ethical leadership and nurse well-being: the mediating role of workplace mindfulness. J. Adv. Nurs. 79, 4008–4021. 10.1111/jan.15719 37226654

[B143] Sison A. G. (2003). The Moral Capital of Leaders: Why Virtue Matters. Cheltenham: Edward Elgar Publishing.

[B144] Snow J. N. Kern R. M. Curlette W. L. (2001). Identifying personality traits associated with attrition in systematic training for effective parenting groups. Fam. J. 9, 102–108. 10.1177/1066480701092003

[B145] Tavakol M. Dennick R. (2011). Making sense of Cronbach's alpha. Int. J. Med. Educ. 2:53. 10.5116/ijme.4dfb.8dfd 28029643 PMC4205511

[B146] Thomas L. B. Wu D. (2006). Expected U.S. budget deficits and the U.S. yield curve. Bus. Econ. 41, 46–53. 10.2145/20060406 17143380

[B147] Treviño L. K. Hartman L. P. Brown M. (2000). Moral person and moral manager: how executives develop a reputation for ethical leadership. Calif. Manage. Rev. 42, 128–142. 10.2307/41166057

[B148] Tushman M. O'Reilly C. A. (2002). Winning Through Innovation: A Practical Guide to Leading Organizational Change and Renewal. Brighton, MA: Harvard Business Press.

[B149] Ullah I. Mirza B. Jamil A. (2021). The influence of ethical leadership on innovative performance: modeling the mediating role of intellectual capital. J. Manag. Dev. 40, 273–292. 10.1108/JMD-08-2020-0277

[B150] Uppathampracha R. Liu G. (2022). Leading for innovation: self-efficacy and work engagement as sequential mediation relating ethical leadership and innovative work behavior. Behav. Sci. 12:266. 10.3390/bs12080266 36004837 PMC9405150

[B151] Valencia Casallas O. L. Barreto-Galeano M. I. (2023). Psychological and institutional factors that predispose to the appearance of corrupt behavior in public and private entities. Psychologia Avances Disciplina 17, 25–42. 10.21500/19002386.6223

[B152] Van Prooijen J. W. (2022). Psychological benefits of believing conspiracy theories. Curr. Opin. Psychol. 47:101352. 10.1016/j.copsyc.2022.101352 35644093

[B153] Walumbwa F. O. Cropanzano R. Goldman B. M. (2011). How leader–member exchange influences effective work behaviors: social exchange and internal–external efficacy perspectives. Pers. Psychol. 64, 739–770. 10.1111/j.1744-6570.2011.01224.x

[B154] Wang C. C. Hsieh H. H. Wang Y. D. (2020). Abusive supervision and employee engagement and satisfaction: the mediating role of employee silence. Pers. Rev. 49, 1845–1858. 10.1108/PR-04-2019-0147

[B155] Wang H. Zhang Y. Li P. Henry S. E. (2023). You raise me up and I reciprocate: linking empowering leadership to organizational citizenship behavior and unethical pro-organizational behavior. Appl. Psychol. 72, 718–742. 10.1111/apps.12398

[B156] Wang L. Xie T. (2023). Double-edged sword effect of flexible work arrangements on employee innovation performance: from the demands–resources–individual effects perspective. Sustainability 15:10159. 10.3390/su151310159

[B157] Wang X. L. Wang M. Y. Liu J. N. (2023). Study on the influence mechanism of leaders' abusive supervision on employees' bootlegging innovation behavior. Int. J. Conflict Manag. 34, 887–906. 10.1108/IJCMA-02-2023-0026

[B158] World Health Organization (2001). The World Health Report 2001: Mental Health: New Understanding, New Hope.

[B159] Yang C. (2014). Does ethical leadership lead to happy workers? A study on the impact of ethical leadership, subjective well-being, and life happiness in the Chinese culture. J. Bus. Ethics 123, 513–525. 10.1007/s10551-013-1852-6

[B160] Yang N. Chen H. Wang X. H. F. (2024). Stealth innovation: the dance of paradoxical leadership behavior, leader trustworthiness, and psychological safety in fueling employee bootlegging behavior. Eur. Manag. J. 1–11. 10.1016/j.emj.2024.03.007

[B161] Yuan L. Chia R. Gosling J. (2023). Confucian virtue ethics and ethical leadership in modern China. J. Bus. Ethics 182, 119–133. 10.1007/s10551-021-05026-5

